# Targeted Radiofrequency Ablation as an Adjunct in Treatment of Lumbar Facet Cysts

**DOI:** 10.7759/cureus.1318

**Published:** 2017-06-06

**Authors:** Jesse Hatgis, Michelle Granville, Aldo Berti, Robert E Jacobson

**Affiliations:** 1 Larkin Hospital, Nova Southeastern University School of Osteopathic Medicine; 2 Miami Neurosurgical Center, University of Miami Hospital

**Keywords:** radiofrequency, radiofrequency ablation, lumbar foraminal stenosis, degenerative spondylolisthesis, lumbar neurogenic claudication, lumbar synovial cyst, lumbar facet cyst, spinal synovial cyst, dorsal epidural cyst

## Abstract

Lumbar facet cysts are frequently found in patients with facet degeneration and segmental instability. When the facet cyst is localized in the neural foramina and lateral recess or becomes large, it can cause radiculopathy or neurogenic claudication. These symptomatic cysts are typically treated interventionally with drainage and a corticosteroid injection or attempts via overinflation to rupture the cyst; however, these procedures have a significant recurrence rate (up to 50%) and often need to be repeated or lead to lumbar surgery if unsuccessful.

This is the first report of using targeted radiofrequency (RF) current as an adjunct to cyst drainage. Although RF has been used for years to treat facet pain indirectly by targeting the medial facet nerve branches, with this technique, under image guidance, the actual cyst is percutaneously drained and then cauterized along with the associated facet capsule, where the original cyst developed. This has improved overall results with less cyst recurrence than previous percutaneous methods and was documented with both intermediate and long-term followup clinically and with magnetic resonance imaging (MRI) scans. This report reviews the underlying anatomy and pathology of the facet joint relating to the development of facet cysts and how current percutaneous treatments for lumbar facet cysts can be supplemented and improved by adding targeted RF ablation to the percutaneous options available to treat a lumbar facet cyst.

## Introduction

The lumbar facet joints are true synovial joints with hyaline cartilage and a highly innervated fibrous capsule. The joints have the capacity to hold 1.0-1.5 mL of fluid [1]. Repetitive stress/trauma results in osteoarthritis that causes inflammation and excess joint fluid, which could stretch the capsule [2]. Chronic degenerative inflammation and stress can lead to the development of cystic deformations from the synovial lining of the joint, and the formation of a symptomatic lumbar facet cyst especially, if it develops from the more ventral surface of the facet joint adjacent to the spinal canal and/or neural foramen. A lumbar synovial facet cyst can be found as an isolated radiologic finding on magnetic resonance imaging (MRI) scans, but more frequently there is often concurrent facet degeneration, spondylolisthesis, or spinal stenosis. When the cyst becomes large or is localized in or around the neural foramen, the cyst can cause radiculopathy or neurogenic claudication. The treatment for symptomatic lumbar facet cysts ranges from simple attempts to inject the cyst with corticosteroids and interventional procedures to drain or rupture the cyst to surgical procedures including microscopic cyst resection, open surgery, and even segmental stabilization, especially if there is associated degenerative spondylolisthesis [3-6]. MRIs are the definitive examination for synovial cysts. Evaluating the fluid characteristics on the MRI scan can help determine if the cyst is easily aspirated or loculated, mucoid, or fibrous. On reviews of axial MRI scans, the facet joint is often wider and contains T2 high intensity signal fluid. Cysts that become fibrous will be less responsive to indirect methods of reducing or bursting the cysts and may ultimately require resection of the cyst or stabilization of the spinal segment [7]. The underlying symptoms, along with any associated lumbar degenerative disease, especially stenosis, spondylolisthesis, and/or instability, are considered in treating both the cyst and preventing later recurrence. The major issue with minimally invasive treatments of lumbar facet cysts is recurrence, which is reported to be as high as 50% for percutaneous procedures including aspirating or bursting the cyst. 

Radiofrequency (RF) rhizotomy has been used in and around the facet joint for pain control by targeting the external branches of the recurrent sensory nerve innervating the facet joint. There are recent reports of using RF heat safely within the epidural space to reduce ligamentous spinal stenosis [8]. This is the first report of a series of cases showing the effectiveness of combining RF current with cyst drainage to lessen the chance of cyst recurrence. The RF current is used to directly cauterize and ablate elements of the lumbar cyst, including the cyst wall and the facet joint capsule from which the cyst originates. The results from this study and long-term followup demonstrate that this technique reduces the frequency of recurrence compared to simple aspiration and steroid injection or cyst rupture.

## Materials and methods

We performed a retrospective chart review of all patients identified with facet cysts on MRI scans within a 36-month period and reviewed their treatments. Patients were evaluated with a combination of imaging techniques, mainly MRIs in conjunction with computerized tomography (CT) scans and flexion/extension radiographs. Radiologic studies determined if the cyst was an isolated finding or part of more extensive lumbar degenerative spondylosis including segmental instability or stenosis.

Combined with physical examination, it was possible to determine if the identified cyst was the main cause of symptoms of low back pain and especially radicular pain or neurogenic claudication. There were a total of 37 patients identified who had lumbar facet cysts on radiologic studies. The decision to treat the cyst either with drainage alone or combined with RF ablation of the cyst, facet joint, and capsule, or open surgery was based on the location and size of the cyst, concurrent lumbar degenerative disease and stenosis, and comorbidities. Associated medical comorbidities such as age, obesity, diabetes, pulmonary and cardiac restrictions, and the use of anticoagulants were all factors in considering the more minimal RF approach versus lumbar surgery. Twenty-four patients had either small facet cysts or cysts associated with more severe stenosis or spondylolisthesis and were not treated with drainage and RF ablation. A subgroup of 13 patients within the total group had either facet cysts or dorsal epidural cysts that were treated with cyst drainage combined with RF ablation of the cyst, facet joint, and capsule.

**Figure 1:**
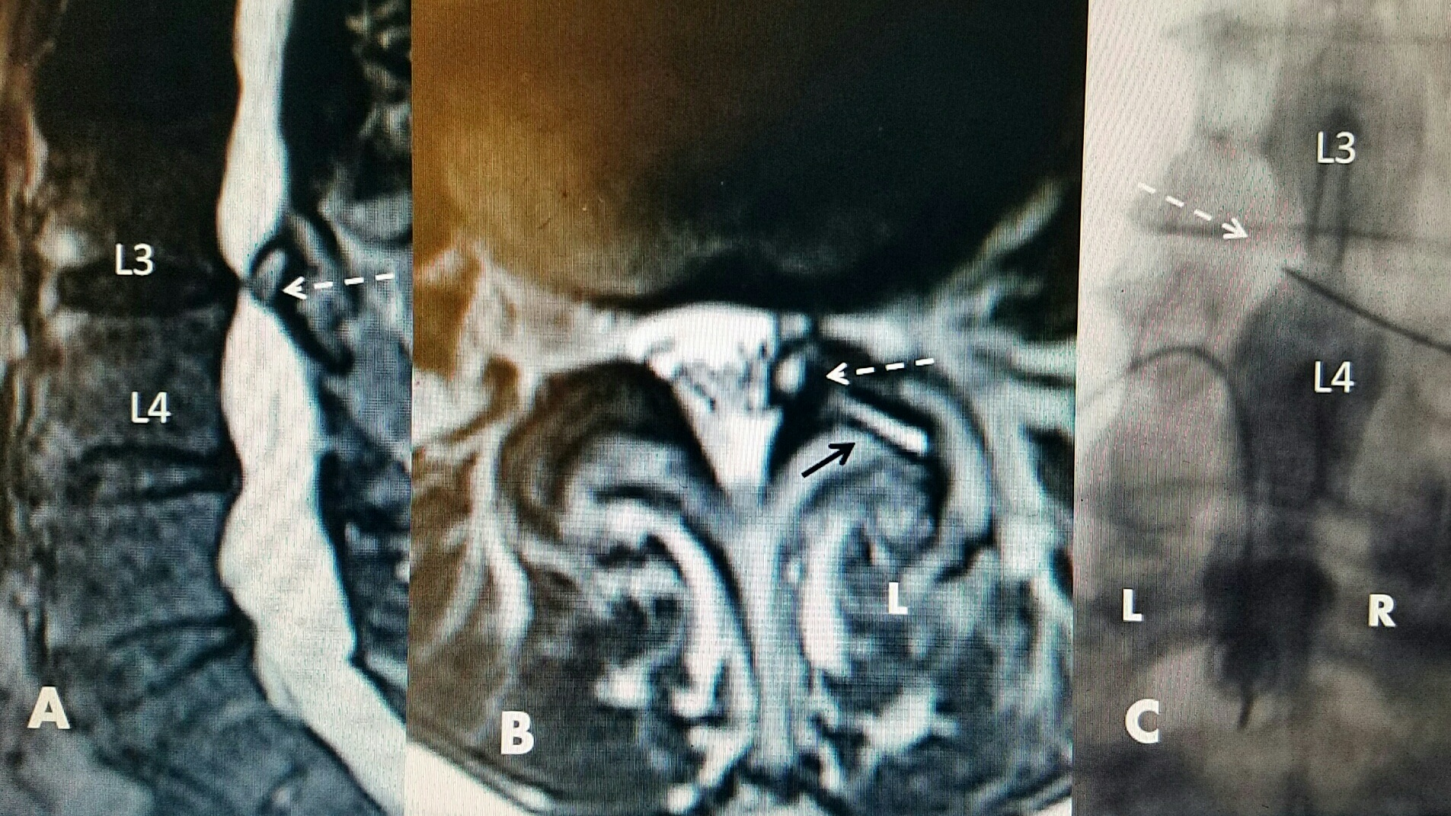
L3-4 facet cyst: magnetic resonance image (MRI) and intraoperative films A: Sagittal T2 spin MRI: localized L3-4 ventrolateral cyst compression (white dashed arrow) causing lateral recess stenosis. B: Axial T2 MRI: left (L) L3-4 facet cyst developing from the ventral surface of the facet joint with high T2 signal intensity (white dashed arrow) causing marked left foraminal stenosis with additional high signal intensity fluid in L3-4 facet joint (black solid arrow) on the same side as facet cyst (white dashed arrow). C: Intraoperative film: left (L) and right (R) are marked as seen in the prone position. Myelographic contrast outlining left L3-4 defect due to the facet cyst (white dashed arrow). The radiofrequency (RF) electrode is entering the defect obliquely through the interlaminar space.

The procedure is performed at an outpatient surgical center under local anesthetic with intra-operative fluoroscopy and minimal sedation as needed. Preoperative antibiotics including either one gram of cephalexin or 600 mg clindamycin is administered intravenously before the procedure. The patient is then placed prone on the procedure table. The skin is sterily cleansed and draped. Lidocaine 1% is used for skin anesthesia. A 20 gauge spinal needle is placed in the intrathecal space to perform a spinal tap, either above or below the targeted level, and a myelogram using 4 to 8 cc of water soluble contrast is performed as the first step of the procedure. The contrast provides precise localization by outlining the cyst corresponding to that seen on the MRI scan. This step allows real-time delineation of the target for RF lesioning (Figure 1). 

**Figure 2:**
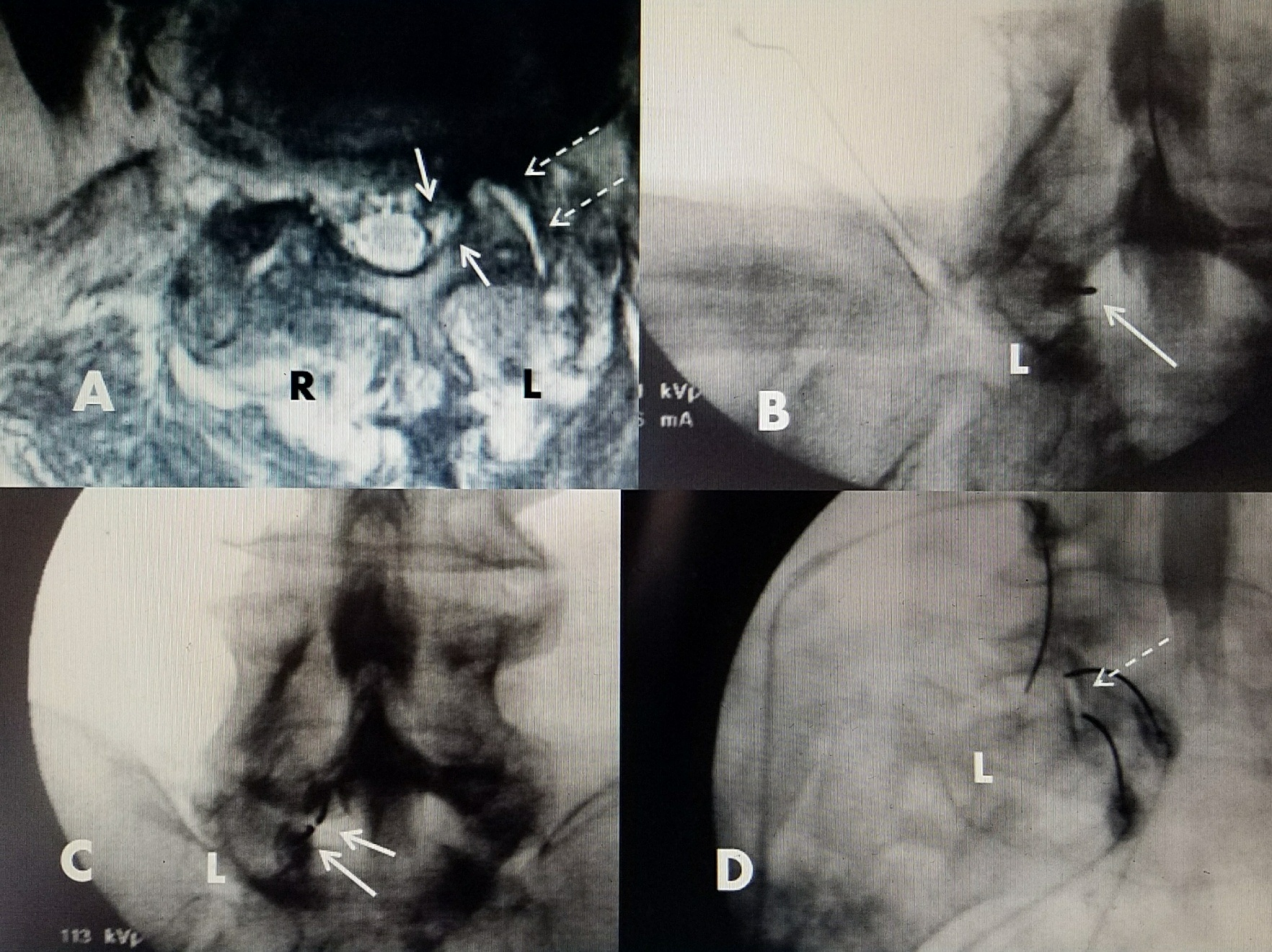
Electrode placement for L5-S1 left facet cyst Left side (L) on intraoperative fluoroscopy. Left side (L) and right side (R) on axial MRI scan. A: Axial T2 MRI showing cyst medial to facet on left side (L) in lateral recess (solid white arrows). High intensity fluid in adjacent facet joint (dashed white arrows). B: Intraoperative film with myelogram contrast showing defect to left (L) corresponding to location of cyst seen on MRI in (A). First RF electrode directed into facet cyst (solid white arrow). C: Second RF electrode placed in cyst below the initial RF electrode (solid white arrows). D: Oblique film showing RF electrodes in L5-S1 facet joint and capsule (dashed white arrows).

Based on the analysis of the preoperative CT, MRI, and intraoperative myelogram, the angle of approach to the cyst can be planned similar to the approach for simple cyst drainage. CT guidance for drainage and RF lesioning can also be performed. Typically, one of three approaches is used. For facet cysts situated in the foramina or lateral recess, a medial to lateral translaminar trajectory with an oblique angle is used to approach the cyst. With this approach, the electrode passes tangentially going away from the dura and posterior to the exiting nerve root to enter the cyst. The other path to a foraminal cyst is a transforaminal approach to target the anterior portion just ventral to the superior articular process of the facet joint and the cyst, which usually is situated ventromedially to the facet [5-6]. More posterior/dorsal cysts are either approached translaminarly, or if there is an obvious posterior interspinous cleft with fluid on the MRI scan, the drainage needle and RF electrodes are passed between the spinous processes through the cleft toward the dorsal cyst. All electrode positions are monitored on anterior-posterior, lateral, and oblique fluoroscopy. Once the introducing needle and cannula are confirmed to be in the cyst, contrast is injected into the cyst. This confirms the exact needle location within the cyst, which is important before RF lesioning. After confirmation of entry into the cyst, the cyst is aspirated. If a spinal needle instead of the RF electrode cannula is used to inject and aspirate the cyst, then a 15 or 20 cm length 20 gauge RF electrode (Stryker® PA, USA) is directed into the cyst. Either an angled or straight 2 or 5 mm exposed tip electrode is placed into the cyst. Next, with the patient awake, sensory and motor testing is performed ranging from 0 to 3 millivolts (mV). If there is a development of an increasing and/or lingering tingling sensation or twitching motion, the electrode is slightly repositioned, usually less than 1-2 mm and testing is repeated. At maximum testing up to 3 mV, when no sensation or movement is present, lesions are sequentially made for 15, 30, and 60 seconds at 60 °C. Sensory and motor testing is repeated with each needle repositioning and before each lesion generation. Following cyst ablation, using oblique fluoroscopy, two or three RF electrodes are then placed in the dorsal facet capsule and, if possible, the electrode tip is threaded into the facet joint itself to ablate the joint capsule at multiple locations using a current set at 80°C for either 60 or 90 seconds. In patients with facet cysts, MRI scans very frequently show additional hyperintense signal fluid in the facet joint on the same side as the cyst. The actual facet joint is also often wider, enabling the RF electrodes to easily enter the actual facet joint space. This enables the RF current to be applied directly within the facet joint. If facet joint fluid is seen bilaterally on the MRI scan, then both facet joints are treated with RF current (figure 2).

After the conclusion of lesioning, all needles are removed, the skin is cleansed, and small bandages are placed overlying the needle insertion sites. Once the patient is returned to the recovery room, ice packs are placed over the insertion sites. Motor and sensory testing is performed before discharge from the ambulatory surgery center. Initial followup is through a phone call the day after the procedure and via an office visit within the first week.

## Results

Thirty-seven patients were identified who had lumbar facet cysts with or without associated lumbar degenerative disease, stenosis, or spondylolisthesis, through MRI and CT scans. Fourteen patients were treated with cyst drainage alone with injection of corticosteroids. Five of these required a repeat procedure for cyst recurrence within six months and had a recurrence rate of 36%. Because of persistent symptoms despite a second attempt to drain the cyst, patients ultimately had lumbar surgery with cyst resection combined with decompression and segmental stabilization. Ten of the original group of 37 patients underwent lumbar surgery as their primary treatment. These patients had MRI findings of small to moderate-sized facet cysts, between 3-5 mm in diameter, associated with spinal stenosis with at least 50% narrowing of the sagittal spinal canal or degenerative spondylolisthesis.

Thirteen patients were identified who underwent drainage combined with RF ablation of the facet cyst and facet joint capsule connected to the cyst. Multiple comorbidities were found, including eight patients with diabetes; therefore, a contraindication to repeated use of steroids existed. Additionally, five patients were obese, three patients had pulmonary or cardiac restrictions, and two were on chronic anticoagulation.

Seven patients had ventrolateral facet cysts and six had cysts that primarily extended into the dorsal epidural space. A repeat RF procedure within six to 12 weeks after the initial drainage and RF treatment was performed in two of 13 patients. One patient only had the cyst treated with RF during the first procedure, and the second patient had the capsule treated but only in one location. During the second treatment, more extensive RF ablation of the same cyst with the inclusion of larger treatment areas of the facet capsule and joint was performed. Fifteen total procedures were performed on 13 patients (Tables 1-2).

**Table 1:** Statistical breakdown of radiofrequency (RF) facet cysts

Radiofrequency (RF) facet cysts: number of patients	7
Patient average age (range 64-88 years)	80.6
Unlateral radicular pain greater than low back pain	6/7
L5-S1 level of stenosis	4/7
L4-5 level of stenosis	3/7
Multilevel stenosis	2/7
Pre-procedure average visual analog scale (VAS) (range 4-9)	6.8
Post-procedure average visual analog scale (VAS) (range 0-4)	1.5

**Table 2:** Statistical breakdown of radiofrequency (RF) interspinous cysts

Radiofrequency (RF) interspinous cysts: number of patients	6
Patient average age (range 54-80 years)	71.5
Unlateral radicular pain greater than low back pain	5/6
Neurogenic claudication	3/6
L4-5 level of stenosis	5/6
L5-S1 level of stenosis	2/6
Multilevel stenosis	3/6
Pre-procedure average visual analog scale (VAS) (average 4-10)	6
Post-procedure average visual analog scale (VAS) (average 0-3)	1.2

For facet cysts, the average age of the patients was 80.6 years. Four patients were male and three were female. Four cysts were at the L5-S1 level and three at the L4-5 level; two of seven had multilevel MRI findings of stenosis or spondylolisthesis at the adjacent spinal segment. Radicular pain was greater than back pain in six of seven, and all seven presented with symptoms unilateral leg pain for less than a year. Pre-procedure average visual analog scale (VAS) was 6.8/10. Post-procedure average VAS at one month was 2/10 and at six months 1.5/10. One patient with a L5-S1 foraminal facet cyst had a repeat procedure within six weeks after a followup MRI demonstrated the same cyst; however, after the second treatment when the facet joint capsule was added to the RF targets, the patient lost the radicular leg pain. No patient developed a recurrent cyst or had later open surgery.

There were six patients who had dorsal cysts with or without interspinous fluid clefts on MRI. Distinct hyperintense T2 signal fluid clefts between the spinous processes were seen on 50% of the MRI scans. The average age was 71.5 and there were five males and only one female. The cysts were located at L4-5 in five of six cases, and two patients had cysts at two levels, both L3-4 and L4-5. There was associated degenerative spondylolisthesis at L4-5 in four cases and there was associated canal stenosis at one or more levels in five of six. One early case had repeat RF because of decreased but persistent symptoms, and the facet capsule and joint were included in the second procedure with further reduction in leg pain. None of the patients with dorsal cysts required open surgery.

*Followup*: The followup period for the patients undergoing drainage plus RF ranged from a minimum of six months to 36 months. Two patients (15%) had repeat procedures but none required open surgery for cyst removal. VAS scores for patients with facet cysts averaged 6.8/10 before the procedure to 1.5/10 at the six-month followup. VAS scores for dorsal cysts averaged 6/10 before and 1.2/10 at the six-month followup. Five patients had a VAS of 0 and a return to full activity. MRI scans were obtained in followup at least one time in nine cases.

In summary, of the total group of 37 patients identified with symptomatic lumbar facet cysts, 10 patients had surgery, including cyst resection as their primary procedure. Fourteen patients underwent only cyst drainage and in five (or 36%) cases the cyst recurred. Of the recurrent cysts, three (or 21%) of the total drainage-only group underwent later surgery. Of the 13 patients that had cyst drainage plus RF ablation of the cyst, facet joint, and capsule, only two (or 15%) had repeat procedures but without any later cyst recurrence after the second procedure. In both patients who needed a second RF procedure, the facet capsule and joint space were not originally targeted with RF. No patient in the RF group, including the two patients who had repeat procedures, underwent later lumbar surgery. 

### Case example

**Figure 3:**
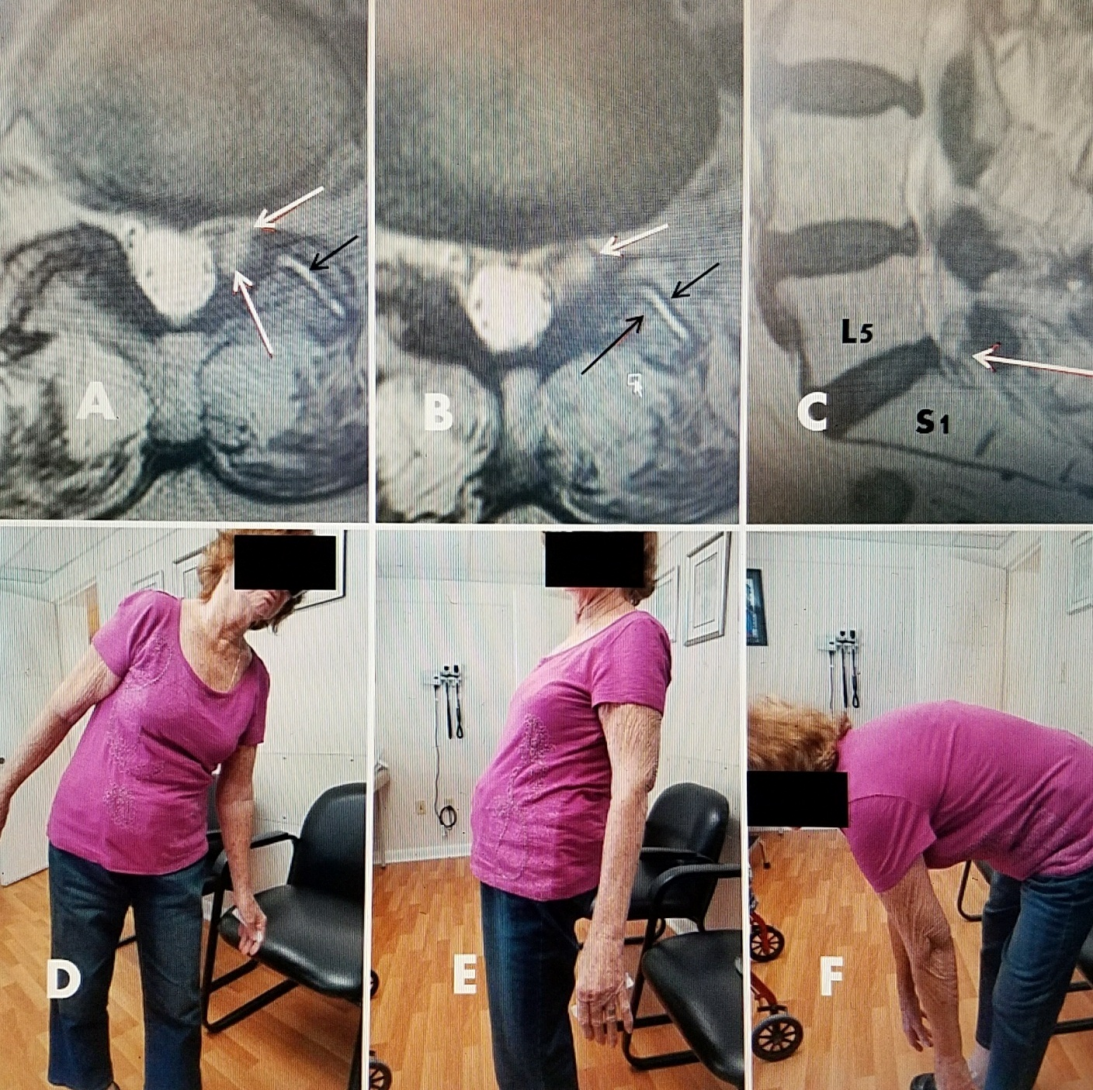
79-year-old female with L5-S1 foraminal cyst with radiculopathy A, B: T2 axial MRI showing left foraminal facet cyst (large solid white arrow) and hyperintense fluid in left facet joint (small black arrow). C: Sagittal T2 MRI showing posterolateral cyst at L5-S1 (white arrow). D: Patient 18 months after drainage and ablation with lateral bending to the left, towards side of cyst. E/F: 18 months after RF cyst ablation. Patient has recovered full movement with extension and flexion without limitation or leg pain.

A 79-year-old female with a history of low-grade lumbar pain for three years subsequently developed, over a period of eight months, increasingly severe and constant left leg pain. This was aggravated by extension and left lateral bending, and it was associated with intermittent left leg numbness. There was no relief with a combination of anti-inflammatory medications and physical therapy. An MRI scan showed a left L5-S1 foraminal cyst. She underwent percutaneous drainage and RF ablation of the cyst and left L5-S1 facet capsule. Pre-procedure VAS was 8/10. Post-procedure she had complete relief of her leg pain and return of full spinal motion. In followup after 18 months, she has continued pain-free with VAS score of 0/10 with a full range of motion (Figure 3).

## Discussion

### Biomechanics and pathophysiology

**Figure 4:**
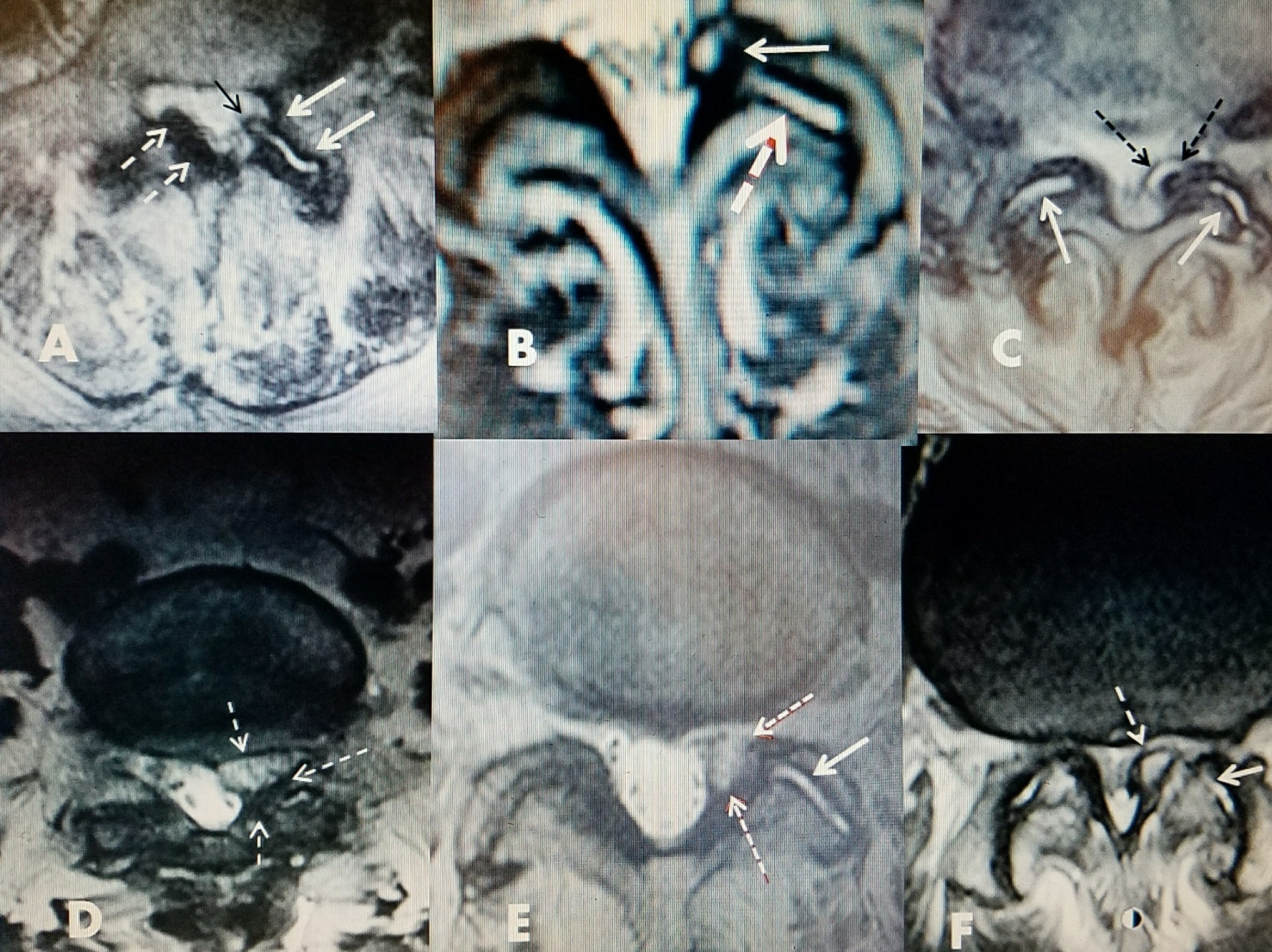
Axial MRI scans showing the different fluid densities within the facet cysts Examples of different MRI characteristics of different lumbar facet cysts. A: Mixture of posterior ligament stenosis (dashed white arrows), development of fluid in facet joint (white arrows), and beginning of cyst on same side extending out of the ventral joint space into the lateral recess (black arrow). B: Unilateral facet and foraminal cyst at L4-5 causing lateral recess stenosis (upper white arrow). High intensity T2 signal change indicates fluid within joint (dashed white arrow). C: Bilateral hyperintense fluid in both facet joints (solid white arrows) shows cyst formation on one side with medial encroachment into lateral and dorsal spinal canal (dashed black arrows). D: Foraminal cyst with more homogenous fluid on MRI that is more gelatinous (dashed white arrows). E: Hyperintense signal of fluid in facet joint (white arrow) and more gelatinous isodense fluid in facet cyst (dashed red/white arrows). F: Fluid in facet joint (white arrow) and bone spur from inferior facet mimicking cyst (dashed arrow).

Repetitive stress/trauma results in osteoarthritis that causes inflammation and joint fluid [8]. This chronic degenerative inflammation and stress, combined with cystic deformation of the synovial lining of the joint, leads to the formation of lumbar facet cysts. The cysts originate from the synovium of the facet joint and frequently remain in continuity. Biomechanical studies have shown that as the normal disc segment degenerates, the mechanical load shifts from the anterior column to the posterior column, which includes both facet joints. It has been experimentally demonstrated that disc degeneration creates a five-fold increased load on the facet joints. Over time, this marked increased load leads to a progressive synovial reaction, and inflammatory fluid formation, which is the basis of the pathophysiology leading to the development of facet cysts. Eventually, there is bone proliferation, facet hypertrophy, and segmental degeneration, instability, and stenosis [9]. Additional chronic hypermobility, secondary to increased joint loading and stretching of the facet capsule ligament, can lead to increased fibrocartilage reaction. This lays the groundwork for the formation of these cysts [9-11]. In a study examining 45 cysts resected during surgery, a communication channel between the cyst and the joint was confirmed in the majority of cases [12]. The involved facet joint also showed moderate to severe degeneration. When the cyst and facet joints were studied pathologically, the cyst walls consisted of elastic and collagen fibers undergoing fibrinoid degeneration, but no synovial lining cells were detected [12]. Associated ligamentous hypertrophy and/or degenerative anterolisthesis adds further compression and stress to the facet joint capsule [13]. If a cystic outpouch forms and the cyst enlarges, it can encroach upon the neuroforamen or spinal canal, often leading to nerve root compression [9, 14-15]. In computer studies of the facet joints, clear deformities in the middle of the facet capsular ligament (FCL) were found. Anterior capsular deformations were more noticeable than posterior ones, as the typical facet cyst develops anteriorly [15]. In large clinical and radiographic series of lumbar facet cysts, the L4-5 level was the most affected (82%) and grade I spondylolisthesis was seen in 47% of cases [16-18]. Examples of different facet cysts with axial MRI studies demonstrate the various sizes and typical ventrolateral location of lumbar facet cysts as well as the associated fluid formation within the adjacent facet joint (Figure 4).

### Treatment

Interventional approaches include intra-articular steroid injections with or without cyst aspiration, medial branch blocks, and epidural blocks with or without attempting to rupture the cyst [16]. Surgeries include cyst removal through microsurgery or tubular resection, ligamentum flavum excision, or laminectomy with or without stabilization. Although percutaneous approaches have less risk and morbidity, surgery has been demonstrated to be the most effective treatment approach [17-18]. Cyst drainage with the concurrent injection of corticosteroids has been the most common initial approach to symptomatic facet cysts; however, there is almost a 50% recurrence of the cysts within the initial six to 12 months after drainage or rupture [19]. In a large series of 101 patients in which 81% of cases had confirmed successful cyst rupture via fluoroscopy, followup showed that 54% of patients later required surgery [20]. Open surgery either through a micro-laminectomy with cyst removal or using a tubular retractor has been reported to have as high as 94% excellent/good results. Patients with spondylolisthesis had a lesser surgical response with 80% good/excellent results [21]. These studies highlight the relationship of the facet cyst to other segmental pathology such as degenerative spondylolisthesis or single and multilevel spinal stenosis. Often, the cyst is a secondary manifestation of broader facet degeneration and instability, and cases with more severe localized stenosis or multilevel bone compression may need lumbar decompression with or without spinal fixation [1, 4]. Since underlying segmental instability with or without degenerative spondylolisthesis is so frequently found in association with symptomatic synovial cysts, cyst resection combined with stabilization is often used with complaints of neurogenic claudication [22]. Another indication that segmental instability is a key component of the problem is a study using interspinous stabilization devices with minimal or no decompression. Ten cases were treated by inserting an interspinous stabilizing device in a minimally invasive approach in patients with facet cysts. Cyst resorption and clinical improvement were reported in half of the patients, and partial improvement was reported in the other half. Interspinous stabilization devices decrease the load and motion of the intervertebral joints, which in turn leads to cyst resorption and symptom improvement [23]. This demonstrates that segmental instability is a major factor both in facet degeneration and facet cyst formation.

### Targeted radiofrequency ablation of lumbar synovial facet cysts and facet joints

In the past, RF facet rhizotomy has been used mainly for pain control without direct targeting of the facet capsule or the facet cyst. In RF rhizotomy, the target is the recurrent medial branch of the sensory nerve innervating the facet capsule. Radiofrequency was used at multiple levels to treat both axial lumbar pain secondary to degenerated facets and referred pain secondary to segmental degeneration, but this does nothing to address the cyst itself or facet joint membrane and capsule [24].

**Figure 5:**
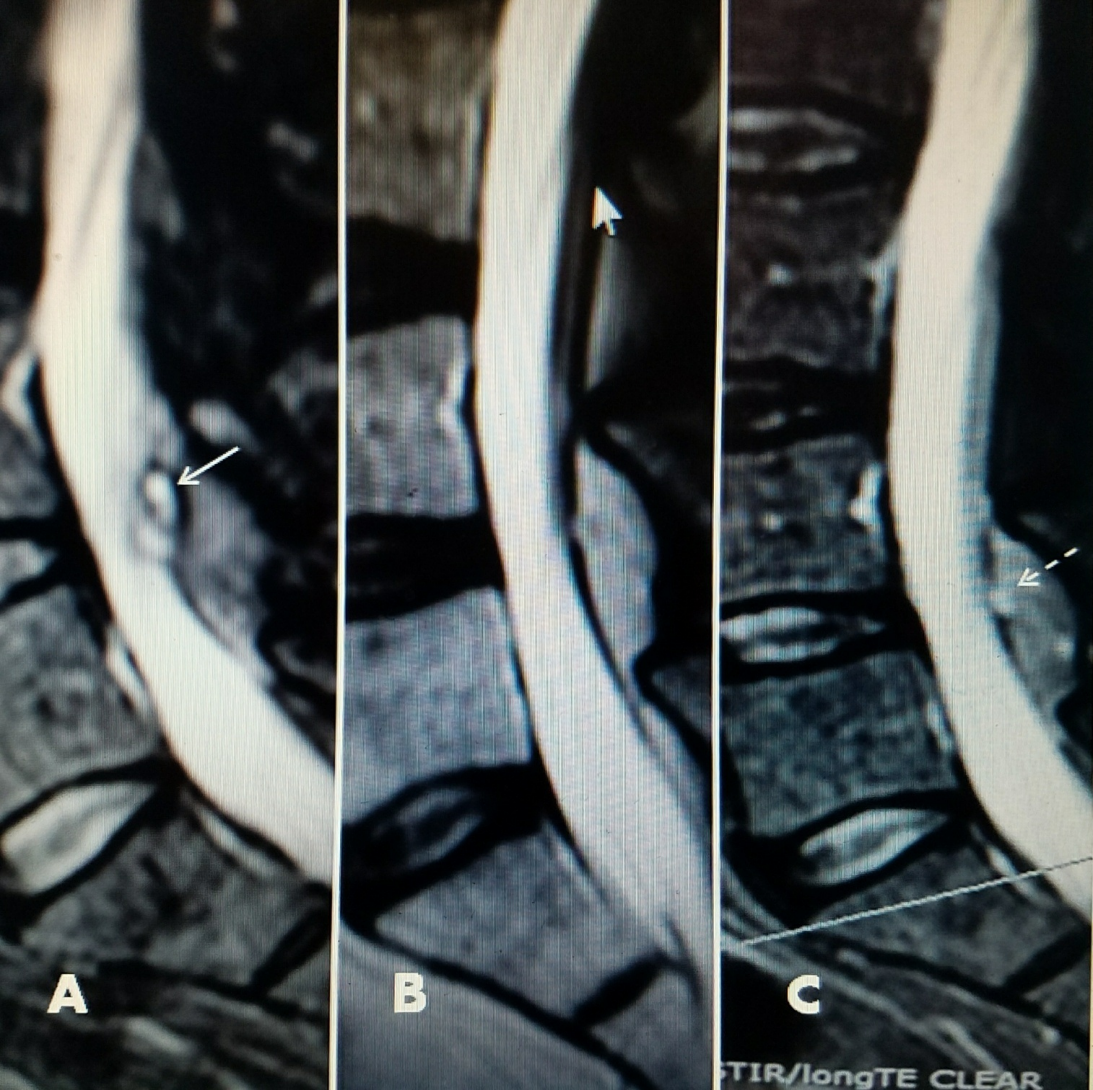
Pre-RF and post-RF followup MRI scans A: Pre-procedure MRI showing 2.2 x 1.4 cm dorsal facet cyst causing lumbar stenosis with neurogenic claudication. Note loculated cystic component (solid white arrow). B: Seven months post drainage and RF ablation of cyst. Patient was asymptomatic.The canal has minimal posterior narrowing. Loculated component disappeared and a smaller dorsal cyst remains. C: Thirty-six months followup MRI. Patient remained asymptomatic. There is microcalcification in cyst (dashed white arrow) but no recurrence of stenosis.

This report describes the use of direct cyst drainage combined with radiofrequency ablation of the facet cyst, the facet capsule, and facet joint. This additional step addresses the original cause of the cyst: the synovial lining of the abnormal facet joint. This approach immediately reduces the cyst and prevents re-accumulation of cyst fluid over a period of time, well beyond the common recurrences within six to 12 months. Clinical response and long-term followup with sequential MRI scans demonstrate the lasting effect of this approach clinically and radiographically over 36 months (Figure 5).

The key underlying pathologic cause of either foraminal or dorsal lumbar facet cysts is the reaction of the facet joint synovial membrane producing inflammatory fluid with accumulation in the joint (as shown by hyper-intense fluid signal on MRI scans) and ultimately cyst development. It was apparent after two of the early cases needed to be repeated that it was important to treat the cyst, the synovium of the facet capsule, and the facet joint to reduce the chance of recurrence. The approach to the cyst and the synovium with RF has not been addressed before this report. Recent studies have shown that RF current can safely be used intra-spinally, around the lumbar nerve roots, and within the contiguous vertebrae as long as the patient can undergo motor and sensory testing [6, 25]. In our longest clinical followup of three years, we confirmed with sequential MRI scans that there was a reduction and stabilization of a large dorsal cyst without re-accumulation or re-expansion. Another important clinical observation in our group of patients, besides the loss of radicular pain or neurogenic claudication after treating the cyst, is the return to full range of spinal movement. This improved spinal mobility allows patients to engage in more advanced physical therapy programs, which in turn promote better spinal strengthening and stabilization [6].

The safety and position of the electrode for RF facet ablation is intraoperatively testable with the patients under local anesthesia. This allows for appropriate patient feedback to avoid any injury to nerve roots as the RF lesion is made. Procedures that only drain or rupture symptomatic synovial cysts have a high recurrence rate, since the synovium is capable of regenerating or re-accumulating fluid within any residual facet cyst. Cysts treated with drainage and RF ablation in the proper locations should have a much lower recurrence rate. 

## Conclusions

Symptomatic lumbar synovial cysts are often effectively treated interventionally with drainage and a corticosteroid injection or overinflation to rupture the cyst; however, this often fails. Cyst drainage and rupture have a significant recurrence rate close to 50% and often need to be repeated. If unsuccessful, the patient usually has open surgery with cyst resection or decompression with or without segmental stabilization. Pathologic studies demonstrate that the underlying cause of cyst formation and the likely reason for frequent recurrence is progressive facet degeneration. This degeneration leads to reactive fluid formation from the synovium of the facet joint and later associated facet joint hypertrophy secondary to segmental instability. 

Combining cyst drainage with cauterizing the facet cyst and joint capsule with RF ablation directly targets one of the primary causes of cyst formation. Use of targeted RF current directly into the cyst as well as the connected facet capsule and joint space is safe with proper intra-procedural testing. In our series of 13 patients, none have had further surgery for removal of the cyst. Serial followup MRI scans in patients have demonstrated lasting reduction in cyst size when comparing them to pre-treatment scans. The average followup was 15 months and the longest followup was 36 months. Clinically, there was a persistent loss of radicular complaints and claudication. There was a marked reduction in post-procedure VAS scores at six months' (or greater) followup. This study demonstrates that combining cyst drainage/rupture with the use of RF current for cauterization/ablation of the facet cyst, its wall, and synovial joint capsule serves as an additional step that can reduce the frequency of cyst recurrence.
